# Impact of Diet on Risk of IBD

**DOI:** 10.1093/crocol/otz054

**Published:** 2020-01-16

**Authors:** Ashwin N Ananthakrishnan

**Affiliations:** Division of Gastroenterology, Massachusetts General Hospital, Boston, MA

**Keywords:** Crohn’s disease, ulcerative colitis, environment, diet, smoking

## Abstract

Diet is an important factor influencing the pathogenesis of Crohn’s disease (CD) and ulcerative colitis (UC). Several recent prospective cohorts have suggested various dietary factors may play a role in modifying the risk of these diseases. These include an inverse association between dietary fiber, fruit or vegetable intake and risk of CD and n-3 polyunsaturated fatty acids and UC. In addition to macro-nutrients, dietary additives such as emulsifiers may also play a role.

Inflammatory bowel diseases (IBD) comprising Crohn’s disease (CD) and ulcerative colitis (UC) affect an estimated 2 million individuals in the United States. While traditionally considered to exclusively affect those of white ancestry residing in Europe and North America, they are increasingly being recognized as global diseases.^1^ Their recent emergence in Asia and South America, regions undergoing rapid industrialization, has drawn renewed attention to the role of environmental determinants of these diseases, prominent among which is diet.^2^ Several layers of evidence of varying robustness support such an association. Globally, the past few decades have seen several key trends in food consumption including decreasing consumption of dietary fiber, increased intake of fats, sugars, and processed foods. The change in these dietary behaviors closely tracks changing incidence of CD and UC worldwide,^3^ and may be particularly relevant in countries which have increasingly seen a westernization of diet distinct from their traditional, diversified diet. Initial examination of dietary risk factors for IBD relied on case–control studies with recall of predisease diet among those with a new diagnosis. This method is particularly fraught with errors in IBD where a predisease period prior to overt symptoms may extend for 2–4 years and individuals frequently make dietary changes following onset of symptoms but prior to diagnosis.

The role of diet in the pathogenesis of IBD has drawn most support from several recent prospective cohorts in North America and Europe that were established primarily to study cancer and cardiovascular disease but have fortuitously been able to shed some important light on the risk of IBD. Analysis of the prospective European Prospective Investigation into Cancer (EPIC) study suggested that a dietary pattern associated with consumption of high sugar and soft drinks and low vegetables had an increase in risk of incident UC when comparing extreme quintiles (OR: 1.68, 95% CI: 1.00–2.82).^4^ However, analysis of the Swedish mammography cohort failed to confirm this association.^5^ The two most consistently (but not universally) replicated dietary associations with IBD are for intake of fiber and dietary fat. In the Nurses Health Study, high intake of dietary fiber was associated with a reduced risk of CD (OR: 0.59, 95% CI: 0.39–0.90).^6^ However, this association differed by fiber source and was strongest for fiber derived from fruits and vegetables, consistent with prior observations.^7^ High intake of dietary n-6 polyunsaturated fatty acids and reduced consumption of marine n-3 PUFA was associated with increased risk of UC in both European and North American cohort studies.^8,9^ Interestingly, this association may be modified by host genetics. Polymorphisms in the cytochrome or fatty acid desaturases modified the association of dietary n-3 or n-6 PUFA intake and risk of UC or CD in two studies.^10^ The association with dietary carbohydrate, protein, dairy, and alcohol is less consistent and most null.

Association with dietary components is not limited to macronutrients alone but also extends to micronutrients and other components of food including preservatives and emulsifiers. In a prospective cohort of women, higher predicted serum vitamin D levels were associated with a lower risk of incident CD. Compared with women with a predicted serum 25-hydroxy vitamin D level < 20 ng/mL, those with a level > 30 ng/mL had a reduced risk of CD (HR: 0.38, 95% CI: 0.15–0.97).^11^ Similarly, higher dietary zinc was associated with reduced risk of CD but not UC.^12^ Polyphenols are components in diet that are proposed to have anti-oxidant properties. In the EPIC cohort, higher dietary intake of resveratrol (OR: 0.40, 95% CI: 0.20–0.82) and flavones (OR: 0.33, 95% CI: 0.15–0.69) was associated with reduced risk of CD.^13^ Although human data are lacking, in animal models, exposure to emulsifiers altered the microbial composition, increased inflammatory cytokines, and resulted in more severe colitis.^14^

There are several mechanisms through which diet may influence intestinal inflammation. It has been well-established that both long-term and short-term dietary intake influences composition of the gut microbiome. Either through wholesale modification of the composition of the microbiome or through encouragement of blooms of specific phyla, diet may contribute to intestinal inflammation. In an elegant experiment, mice fed a high milk fat diet developed a bloom of a deltaproteobacteria—*Bilophila wadsworthia—*that was associated with development of colitis.^15^ Second, various dietary components may play a role in maintaining the integrity of the epithelial barrier. In cell culture models, addition of soluble fiber to the medium reduced bacterial translocation across the membrane.^16^ Zinc has also been proposed to contribute to the integrity of the epithelial barrier, and through it, prevent relapses of IBD. Finally, dietary components or ligands found in foods can directly interact with the immune system. For example, cruciferous vegetables contain ligands for the aryl hydrocarbon receptor which plays an anti-inflammatory role.^17^ Administration of vitamin D supplementation ameliorates colitis in vitamin D receptor-knock out mice through the direct effects of vitamin D on innate immune response pathways.

There are several future directions for research into the role of diet in IBD. While providing valuable directions for future investigation and confirmation of effect, existing prospective cohort studies have important limitations. The prospective studies have all been conducted in North America and Europe and are thus applicable to a predominantly white population eating a western diet. Whether similar associations exist for varying dietary habits is unclear. They also investigate primarily IBD that has an onset at an older age. While diet consumed in high school may also potentially influence disease risk, the relative impact of diet on pediatric onset IBD is unclear. Importantly, while these studies are robust for assessing the association with macro- or micronutrient intake, they lack the ability to granularly capture other potentially relevant variables including method of food processing and added preservatives and emulsifiers which could also play a role. The role of dietary factors in mediating recurrence of inflammation in patients with IBD remains to be robustly established, limited by lack of prospective cohorts to address this. There also needs to be an investigation, as outlined above, of not just how host genetics may influence susceptibility or risk conferred by specific dietary components, but how an individuals’ baseline microbiome may confer resistance to the effect of specific dietary components. In summary, the role of dietary factors in the development or propagation of IBD is among the most common questions a provider is asked by their patient and while we may not have a final answer on that yet, we can be reassured by the pace of emerging clinical and translational research in this area that will inform our practice in the near future.

Funding: A.N.A is supported by funding from the Crohn’s and Colitis Foundation and the Chleck Family Foundation.

Conflicts of interest: None.
